# Variation in missed doses and reasons for discontinuation of anti-tuberculosis drugs during hospital treatment for drug-resistant tuberculosis in South Africa

**DOI:** 10.1371/journal.pone.0281097

**Published:** 2023-02-13

**Authors:** Elize Pietersen, Kim Anderson, Helen Cox, Keertan Dheda, Aihua Bian, Bryan E. Shepherd, Timothy R. Sterling, Robin M. Warren, Yuri F. van der Heijden

**Affiliations:** 1 Department of Medicine and UCT Lung Institute & South African MRC/UCT Centre for the Study of Antimicrobial Resistance, Division of Pulmonology, Centre for Lung Infection and Immunity, University of Cape Town, Cape Town, South Africa; 2 Centre for Infectious Disease Epidemiology and Research, School of Public Health and Family Medicine, University of Cape Town, Cape Town, South Africa; 3 Wellcome Centre for Infectious Diseases Research in Africa and the Institute of Infectious Disease and Molecular Medicine, University of Cape Town, Cape Town, South Africa; 4 Faculty of Infectious and Tropical Diseases, Department of Infection Biology, London School of Hygiene and Tropical Medicine, London, United Kingdom; 5 Department of Biostatistics, Vanderbilt University School of Medicine, Nashville, Tennessee, United States of America; 6 Vanderbilt Tuberculosis Center, Vanderbilt University School of Medicine, Nashville, Tennessee, United States of America; 7 Department of Medicine, Division of Infectious Diseases, Vanderbilt University School of Medicine, Nashville, Tennessee, United States of America; 8 Division of Molecular Biology and Human Genetics, NRF-DSI Centre of Excellence for Biomedical Tuberculosis Research, South African Medical Research Council Centre for Tuberculosis Research, Stellenbosch University, Cape Town, South Africa; 9 The Aurum Institute, Johannesburg, South Africa; University of Oxford, UNITED KINGDOM

## Abstract

**Background:**

Updated World Health Organization (WHO) treatment guidelines prioritize all-oral drug-resistant tuberculosis (DR-TB) regimens. Several poorly tolerated drugs, such as amikacin and para-aminosalicylic acid (PAS), remain treatment options for DR-TB in WHO-recommended longer regimens as Group C drugs. Incomplete treatment with anti-TB drugs increases the risk of treatment failure, relapse, and death. We determined whether missed doses of individual anti-TB drugs, and reasons for their discontinuation, varied in closely monitored hospital settings prior to the 2020 WHO DR-TB treatment guideline updates.

**Methods:**

We collected retrospective data on adult patients with microbiologically confirmed DR-TB between 2008 and 2015 who were selected for a study of acquired drug resistance in the Western Cape Province of South Africa. Medical records through mid-2017 were reviewed. Patients received directly observed treatment during hospitalization at specialized DR-TB hospitals. Incomplete treatment with individual anti-TB drugs, defined as the failure to take medication as prescribed, regardless of reason, was determined by comparing percent missed doses, stratified by HIV status and DR-TB regimen. We applied a generalized mixed effects model.

**Results:**

Among 242 patients, 131 (54%) were male, 97 (40%) were living with HIV, 175 (72%) received second-line treatment prior to first hospitalization, and 191 (79%) died during the study period. At initial hospitalization, 134 (55%) patients had *Mycobacterium tuberculosis* with resistance to rifampicin and isoniazid (multidrug-resistant TB [MDR-TB]) without resistance to ofloxacin or amikacin, and 102 (42%) had resistance to ofloxacin and/or amikacin. Most patients (129 [53%]) had multiple hospitalizations and DST changes occurred in 146 (60%) by the end of their last hospital discharge. Incomplete treatment was significantly higher for amikacin (18%), capreomycin (18%), PAS (17%) and kanamycin (16%) than other DR-TB drugs (P<0.001), including ethionamide (8%), moxifloxacin (7%), terizidone (7%), ethambutol (7%), and pyrazinamide (6%). Among the most frequently prescribed drugs, second-line injectables had the highest rates of discontinuation for adverse events (range 0.56–1.02 events per year follow-up), while amikacin, PAS and ethionamide had the highest rates of discontinuation for patient refusal (range 0.51–0.68 events per year follow-up). Missed doses did not differ according to HIV status or anti-TB drug combinations.

**Conclusion:**

We found that incomplete treatment for second-line injectables and PAS during hospitalization was higher than for other anti-TB drugs. To maximize treatment success, interventions to improve person-centered care and mitigate adverse events may be necessary in cases when PAS or amikacin (2020 WHO recommended Group C drugs) are needed.

## Introduction

After SARS-CoV-2, tuberculosis (TB) is the deadliest infectious disease globally and most TB high-burden countries are not on track to reach World Health Organization (WHO) End TB Strategy targets [1]. Drug-resistant TB (DR-TB) poses further public health challenges due to increased duration of treatment, exorbitant costs, more toxic medications and worse patient outcomes than drug-susceptible TB (DS- TB) [1–5]. The treatment success rate for rifampicin-resistant TB (RR-TB) and multidrug-resistant TB (MDR-TB; resistance to at least isoniazid and rifampicin) was 59% globally and 69% in the Africa region in 2018 [1].

South Africa has among the highest global incidence rates of DS-TB and DR-TB [1,6]. In 2011 South Africa implemented a policy of decentralized and deinstitutionalized care for DR-TB patients that shifted care of DR-TB to the outpatient, primary care level [7]. In this system, patients with severe clinical disease or with extensively drug-resistant TB (XDR-TB) may still be hospitalized. One study found that after decentralization, 43% of 2,878 patients with RR-TB in the Western Cape Province of South Africa had evidence of hospitalization for TB within one year of initial RR-TB diagnosis [8].

Adherence to anti-TB drugs for the treatment of both DS-TB and DR-TB is essential for successful treatment and to prevent *Mycobacterium tuberculosis* transmission [9–11]. Non-adherence, including incomplete or irregular adherence to TB medications, increases the risk of death, relapse and acquired drug-resistance [12–16]. Adherence to <90% of prescribed doses was the most significant risk factor for unfavorable outcomes in a pooled analysis of DS-TB treatment shortening trials, and TB treatment adherence has been sub-optimal even in some clinical trial settings [17–19].

Directly-observed therapy (DOT) has been a long-standing strategy to improve adherence to TB treatment [20]. Studies of DOT effectiveness for TB treatment completion or improving treatment outcomes have had variable results, however, and patient-centered approaches including self-administered therapy with community-based support have been proposed [21–23]. In some settings, patients known to be non-adherent may be hospitalized to improve adherence and ultimately treatment success, and to prevent community *M*. *tuberculosis* transmission [24]. Adherence to TB medications during hospitalization has not been widely reported, and it is not known how patient-initiated reasons (e.g., medication refusal) or other factors (e.g., medication intolerance) contributed to incomplete treatment with TB drugs during hospitalization, particularly for DR-TB patients. We sought to characterize missed doses of individual anti-TB drugs, and reasons for their discontinuation, in hospitalized patients with DR-TB.

## Methods

### Study design and patient cohort

**Fig 1:**
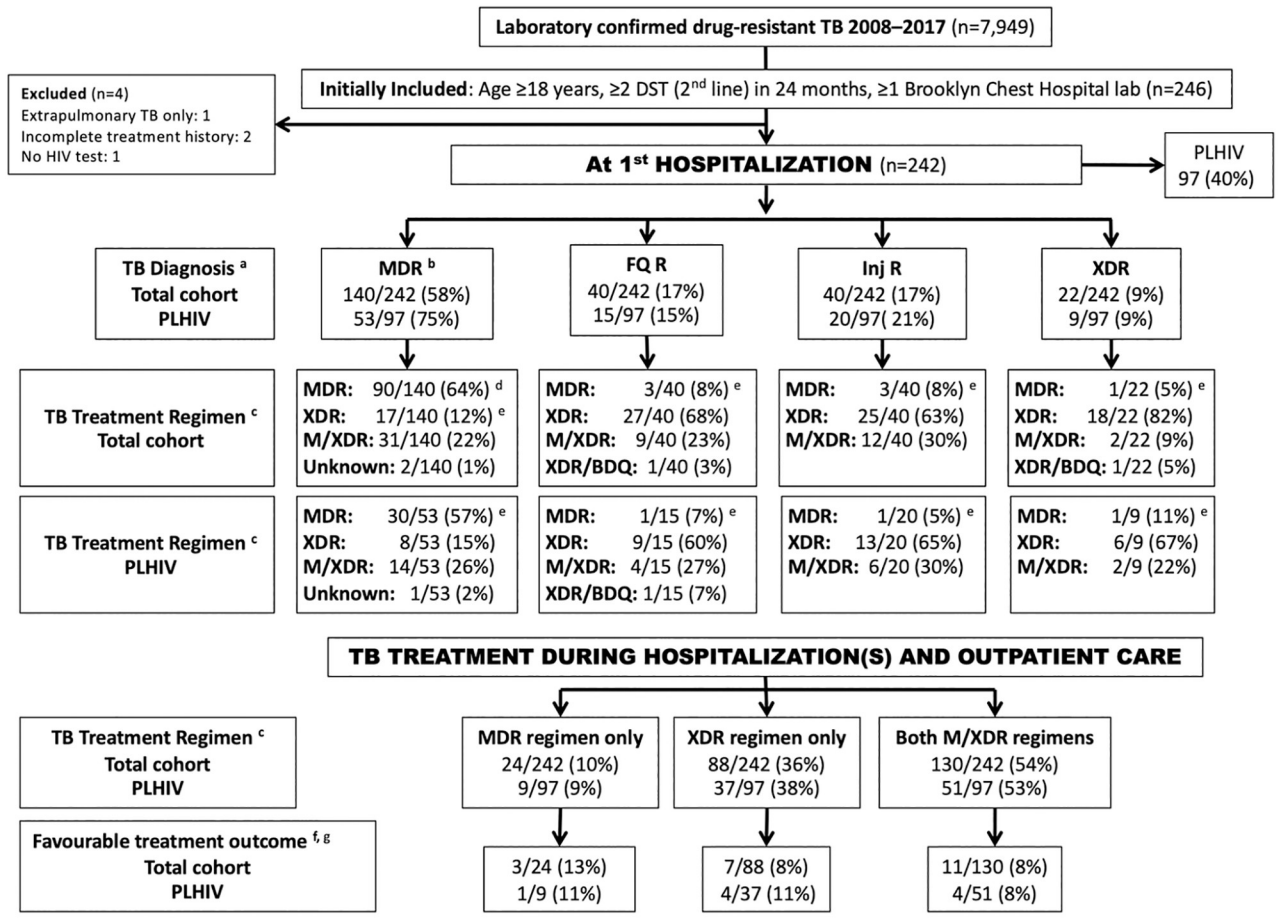
Study profile of patient cohort. Stratified by HIV status, with laboratory confirmed drug-resistant TB, regimens received and treatment outcomes. ^a^ TB Diagnosis: MDR: Resistance to isoniazid and rifampicin; FQ R: Resistance to isoniazid and rifampicin and a fluoroquinolone; Inj R: Resistance to isoniazid and rifampicin and a second-line injectable drug (i.e., amikacin, capreomycin or kanamycin); XDR: Resistance to isoniazid and rifampicin, a fluoroquinolone and a second-line injectable drug. ^b^ Includes 6 mono-resistant patients [isoniazid resistant, rifampicin susceptible (n = 4); rifampicin resistant, isoniazid susceptible (n = 2)] diagnosed with DR-TB during study period. ^c^ MDR regimen included kanamycin; XDR regimen included capreomycin and/or PAS; M/XDR regimen: Treated with a MDR and XDR regimen; XDR/BDQ regimen: Treated with an XDR regimen and bedaquiline added. ^d^ Includes 1 patient treated with isoniazid, rifafour (fixed-dose combination of isoniazid, rifampicin, pyrazinamide, and ethambutol), streptomycin. ^e^ Diagnosed MDR-TB: XDR regimen prescribed for patients in whom MDR regimen failed; Diagnosed FQ R or Inj R or XDR: MDR regimen prescribed in DR-TB treatment naïve patients. ^f^ Combination of Cure and Treatment completed. ^g^ Outcome of XDR/BDQ treatment regimen not shown. DST: Drug-susceptibility test. PLHIV: People living with HIV.

We studied retrospective clinical data of adult (age ≥18 years) patients, with microbiologically confirmed DR-TB between 2008 and 2015, who were selected for a study of acquired anti-TB drug resistance in the Western Cape Province of South Africa (Fig 1).

Laboratory and healthcare facility records were used to capture clinical data and vital status (dead, alive, presumed alive), through July 31, 2017, in REDCap (Research Electronic Data Capture), a secure web-based software platform designed to support data capture for research studies [25,26]. Vital status was verified with two independent external databases: the Western Cape Provincial Health Data Centre and the National Population Register. The study and waiver of informed consent were approved by the Human Research Ethics Committees of the University of Cape Town (HREC 614/2014) and Stellenbosch University (REF N14/08/106), the Vanderbilt University Institutional Review Board (IRB 131289), and City of Cape Town and Western Cape Province authorities.

### Drug-resistant TB diagnosis

The drug susceptibility test (DST) result on or closest before the date of the first hospitalization was recorded as the TB diagnosis at the initial hospitalization. DST changes that occurred during a single or multiple hospitalization periods or during subsequent community-based treatment within the same treatment period were recorded. If more than one DST change occurred during hospitalization the result closest before the date of discharge was recorded. DST changes after the last discharge date were not recorded.

We used definitions of *M*. *tuberculosis* drug resistance applicable during the study period. Any diagnosis of MDR-TB (defined as resistance to at least isoniazid and rifampicin), pre-XDR-TB (defined as resistance to isoniazid and rifampicin and either a fluoroquinolone or a second-line injectable drug but not both) or XDR-TB (defined as MDR-TB plus resistance to a fluoroquinolone and second-line injectable drug) were considered drug-resistant TB (DR-TB).

### Treatment

DR-TB treatment was prescribed according to South African National TB programme guidelines [7], which were based on WHO recommendations. The guidelines included standardized regimens for adult MDR-TB and XDR-TB treatment which could be modified by the treating provider based on DST results and clinical presentation (S1 Table in S1 File). For some of the treatment regimens, providers included anti-TB drugs recommended for DS-TB treatment: rifampicin, streptomycin and/or Rifafour (fixed-dose combination of isoniazid, rifampicin, pyrazinamide, and ethambutol).

For this study we classified DR-TB regimens as MDR, XDR or M/XDR. MDR regimens included kanamycin or amikacin while XDR regimens included capreomycin and/or para-aminosalicyclic acid (PAS) as core anti-TB drugs. Limited alternative treatment options existed in terms of core anti-TB drugs to include in a regimen due to programmatically available drugs and high rates of background and individual drug-resistance in the study population. The designation of M/XDR regimen indicated that patients were treated with both MDR and XDR regimens within the same treatment period. Missed doses of bedaquiline (BDQ), linezolid, pretomanid or delamanid were recorded when prescribed as part of or following an XDR or M/XDR regimen. Some patients, for whom drug-resistant TB treatment failed, met the criteria to be included in the BDQ clinical access programme [27] or the Nix-TB study [28]. For these patients, missed doses were reported for drugs included in their drug-resistant TB regimens. Missed doses of drugs included as part of their BDQ clinical access programme or the Nix-TB study program were not reported due to incomplete or uncertain treatment-related data.

### Hospitalization

All patients were hospitalized at Brooklyn Chest Hospital (BCH) and some patients were also hospitalized at DP Marias Hospital (DPM), two specialized DR-TB hospitals in Cape Town, Western Cape Province, South Africa. We reported missed doses only for those hospitalizations in BCH and/or DPM with available prescription charts. Missed doses during hospitalization at other facilities which administered DR-TB treatment was recorded when available. Missed doses were not assessed when a patient was hospitalized for the exclusive purpose of palliative care.

### Incomplete treatment

We defined complete treatment as taking a course of anti-TB medications as prescribed [17]. Incomplete treatment referred to missed doses of anti-TB medications. We used receipt of <90% of doses of an individual anti-TB drug as a cutoff to define incomplete treatment [19,29], regardless of the reason. Reasons included both active choices made by patients and other reasons, such as intolerance of a drug.

During hospitalizations, nursing staff documented administration of each individual anti-TB drug on a patient’s prescription chart. Researchers recorded the number of doses documented as received and expected to be given based on prescription chart data. Percent received doses was determined for each individual drug by dividing the combined number of doses received by the number of doses prescribed, expressed as a percentage. We calculated percent missed doses (the complement to percent received doses) for each drug by dividing the combined number of missed doses by the number of doses prescribed, expressed as a percentage.

Number of doses from available prescription charts were added across hospitalizations if they were part of the same treatment period. For patients who received multiple courses of the same drug during one or more hospitalizations, we combined treatment data to report a single percentage reflecting doses received per drug per patient.

When discrepancies occurred between prescription chart elements (e.g., between physician prescription orders and information recorded on prescription charts), we sought evidence in the remainder of the medical record to determine the correct information. If discrepancies remained, we captured what was recorded on the prescription charts.

Nursing staff used four standard numerical codes, applicable to prescription charts across all provincial hospitals in the Western Cape Province, to indicate whether doses were missed due to 1) patient refusal, 2) patient away from the ward, 3) anti-TB drug out of stock or 4) patient intolerant to drug. All four standard numerical codes were recorded as drug not received.

In cases where prescription chart(s) were missing from a folder, or a gap of >7 consecutive days occurred between documented administration of a drug, recording of missed doses was stopped on the last day a drug was indicated as received (considered ‘discontinued’), and resumed as a separate ‘course’ if/when there was evidence of continued administration of the drug.

Reasons for discontinuation of individual drugs during hospitalization or at discharge were independently assessed from reasons for missed doses. Reasons for discontinuation of individual drugs were: patient refusal of treatment, patient premature departure from hospital, adverse event, death, treatment failure (as noted by the treating physician), course of treatment completed, changed to another anti-TB drug, continued as community-based treatment, transferred to another facility or province, treatment continued beyond censor date, granted leave from hospital, treatment interruption of >7 days, uncategorized, and unknown. These reasons were extracted from prescription charts or elsewhere in the medical folder.

High rates of background and individual anti-TB drug resistance limited treatment options in our study patients, particularly those who experienced adverse events. We performed a sensitivity analysis for drugs that were discontinued due to adverse events. In this analysis we calculated missed doses by adding the remainder of the intended treatment duration to the doses prescribed (denominator). We determined the intended treatment duration for a drug discontinued due to adverse events by combining courses of the drug prescribed in the same treatment episode, whilst considering the dosing frequency, the duration recommended per guidelines for the drug, and avoiding duplicate doses prescribed when a drug re-challenge was documented.

Treatment data for cycloserine were incorporated with terizidone.

### Data analysis

We reported clinical characteristics of study patients using counts and percentages for categorical variables and medians and interquartile ranges (IQR) for continuous variables. We compared clinical characteristics and percent missed doses by HIV status using the chi-squared test for categorical variables and the Wilcoxon rank-sum test for continuous variables. We evaluated clinical variables at the time of first hospitalization. We assessed percent missed doses per anti-TB drug during hospitalizations, evaluated reasons for anti-TB drug discontinuation, and stratified analyses by HIV status, number of hospitalizations, and DR-TB regimen. We performed data quality review for over half of study participants by duplicate chart review of key treatment-related data fields.

We used a generalized logistic mixed effects model to estimate adjusted odds ratios (OR) and 95% confidence intervals (CI) of receiving a drug (versus missing a drug) during hospitalization. The model included as exposure variables the twelve drugs prescribed most frequently for MDR-TB or XDR-TB in our dataset and for which the most compelling data exist for inclusion in TB treatment regimens: amikacin, capreomycin (reference drug), clofazimine, ethambutol, ethionamide, isoniazid, kanamycin, moxifloxacin, ofloxacin, PAS, pyrazinamide, and terizidone. We did not include amoxicillin, dapsone, or macrolide antibiotics. The model adjusted for HIV status at the time of first hospitalization to measure the association between HIV status and missed doses of each drug, controlling for other anti-TB drugs. The model included a random intercept per patient to account for correlation due to multiple doses per patient. To determine whether percent received doses of individual drugs differed from earlier to later hospitalizations, we assessed the association between patients’ hospitalization number for DR-TB treatment (outcome, categorized as first, second, third, and ≥fourth hospitalization because few patients had more than 3 hospitalizations) and received doses of each medication during that hospitalization (exposure, continuous) using a proportional odds model with robust sandwich variance estimator.

We evaluated factors associated with percent received doses of PAS using a cumulative probability model on a subset of study patients who were treated with PAS at any time during the study [30,31]. We included sex, age at first DR-TB admission, race, HIV status, and history of previous TB to determine the association between each covariate and missed doses of PAS. Analyses used two-sided P values and P<0.05 was considered significant. We performed analyses using Stata (version 15.1; StataCorp, College Station, TX, USA) and R statistical software (version 3.6.3; R Foundation for Statistical Computing, Vienna, Austria).

## Results

Among 242 patients with DR-TB, median age was 34 years (IQR 26, 42) at first hospitalization, 131 (54%) were male, 146 (60%) had a previous history of smoking, and 168 (69%) had a history of substance use (Table 1).

**Table 1:** Patient characteristics. Characteristics of 242 hospitalised drug-resistant tuberculosis patients stratified by HIV status. Data are median (interquartile range) or n/N (%) unless otherwise stated.

	HIV negative N = 145 (60)	HIV positive N = 97 (40)
**Demographic characteristics**		
Age at 1^st^ hospitalization, years^a^	34 (25, 43)	34 (29, 39)
Male Sex	91 (63)	40 (41)
Race		
Mixed ancestry	106 (73)	33 (34)
Black	34 (23)	63 (65)
Other (Caucasian, Indian)	5 (3)	1 (1)
Smoking ever: Yes	100/138 (72)	46/93 (49)
Substances ever: Yes	110/144 (76)	58/95 (61)
Weight at 1^st^ hospitalization, kg^a^	50.0 (43.9, 55.7) (n = 127)	49.4 (45.0, 57.5) (n = 93)
Education		
None	2 (1)	5 (5)
Primary	42 (29)	21 (22)
Secondary	88 (61)	59 (61)
Tertiary	6 (4)	3 (3)
Unknown	7 (5)	9 (9)
**Clinical characteristics**		
HIV-positive at 1^st^ hospitalization	-	96/97 (99)
On anti-retroviral therapy	-	92/96 (96)
CD4 count, cells/μl^a^ (n = 93)	-	281 (118, 380)
Chest X-ray done at 1^st^ hospitalization	75 (52)	49 (51)
Zero score (no lung disease or cavity)	6/75 (8)	2/49 (4)
Total score (lung disease plus cavity)^a^	6.75 (4, 8.75)	5.5 (4.5, 7.25)
Previous DS-TB^b^	102 (70)	78 (80)
Chronic kidney disease	4 (3)	3 (3)
Diabetes	6 (4)	2 (2)
Mental health diagnosis	12 (8)	2 (2)
**Number of hospitalizations for drug-resistant TB (DR-TB)** ^c^		
Single hospitalization	61 (42)	52 (54)
Multiple hospitalizations	84 (58)	45 (46)
Duration of hospitalization in TB hospitals, days^a^	413 (242, 566)	339 (229, 541)
**Overall TB treatment received during hospitalization(s) and outpatient** (See Fig 1)		
MDR^d^	15 (10)	9 (9)
XDR^e^	51 (35)	37 (38)
M/XDR (MDR plus XDR)	79 (54)	51 (53)
**DR-TB diagnosis at 1**^**st**^ **hospitalization**		
Mono-resistant (isoniazid or rifampicin)	6 (4)	0
MDR-TB^f^	81 (56)	53 (55)
Pre-FQ-TB^g^	25 (17)	15 (15)
Pre-Inj-TB^h^	20 (14)	20 (21)
XDR-TB^i^	13 (9)	9 (9)
**Vital Status at end of study**		
Confirmed alive	22 (15)	11(11)
Presumed alive	11 (8)	7 (7)
Deceased	112 (77)	79 (81)

^a^ Median (Interquartile range).

^b^ DS-TB: Drug susceptible TB.

^c^ Same day transfer between specialist DR-TB hospitals considered as one hospitalization.

^d^ MDR regimen: Included kanamycin or amikacin as core TB drug.

^e^ XDR regimen: Included capreomycin and/or para-aminosalicyclic acid (PAS) as core anti-TB drug.

^f^ MDR-TB: Defined as resistance to at least isoniazid and rifampicin.

^g^ Pre-FQ-TB: Defined as resistance to isoniazid and rifampicin and a fluoroquinolone.

^h^ Pre-Inj-TB: Defined as resistance to isoniazid and rifampicin and a second-line injectable drug.

^i^ XDR-TB: Defined as MDR-TB plus resistance to a fluoroquinolone and second-line injectable drug.

A total of 96 (40%) were known to be people living with HIV (PLHIV) at the time of their first hospitalization for DR-TB, 96% of whom were treated with antiretroviral therapy (ART). One additional patient was diagnosed HIV-positive during the study period and was started on ART. For 51 PLHIV with a known CD4 count in the 12 months preceding or in the first week of first hospitalization, the median CD4 count was 207 cells/μl (IQR 63, 331). Median follow-up time was 2.32 years. By the end of the study period, 191 (79%; 95% confidence interval [CI] 73–84%) patients had died. Overall, 81/191 (42%) patients died while hospitalized. During their first hospitalization 31/81 (38%) patients died and 33/81 (41%) during 2^nd^, 9/81 (11%) during 3^rd^, and 8/81 (10%) during 4^th^ to 6^th^ hospitalizations.

### Drug-resistant TB diagnosis

Most patients (171 [71%; 95% CI 65–76%]) were diagnosed with DR-TB within 6 months prior to their first hospitalization for DR-TB, 60 (25%; 95% CI 20–31%) were diagnosed more than 6 months prior to first hospitalization and 11 (5%; 95% CI 3–8%) were diagnosed at the time of hospitalization. TB diagnoses at first hospitalization included 6 (2%; 95% CI 1–5%) with isoniazid or rifampicin mono-resistance, 134 (55%; 95% CI 49–62%) with MDR-TB without additional resistance, 80 (33%; 95% CI 27–39%) with pre-XDR-TB (MDR-TB with resistance to ofloxacin or amikacin but not both), and 22 (9%; 95% CI 6–13%) with XDR-TB (Fig 1). Most patients (129 [53%; 95% CI 47–59%]) had ≥2 TB-related hospitalizations during the study period. Among all patients, 146 (60%; 95% CI 54–66%) acquired additional resistance during the treatment period up to discharge from the last hospitalization. In patients who had a single hospitalization, the DST profile of 57/113 (50%) patients changed.

### Drug-resistant TB treatment

Among all patients, capreomycin, pyrazinamide, terizidone, ethambutol, and PAS were the most frequently prescribed anti-tuberculosis drugs. MDR treatment included a median of 5 (IQR 5, 6) drugs and XDR treatment 9 (IQR 7, 9) drugs. The higher number of drugs in XDR treatment episodes reflects the common addition of drugs such as amoxicillin, amoxicillin-clavulanate, and clarithromycin (each of which were reported separately) (S1 Table in S1 File).

### Hospitalizations

The total duration of hospitalization in TB treatment hospitals was a median of 399 days (IQR 235, 562). Median duration of hospitalization was 339 days for HIV-positive and 413 days for HIV-negative patients (P = 0.28). The majority of patients had multiple hospitalizations (129 [53%]) with most hospitalized twice (83/129 [64%]) or three times (23/129 [18%]) during one treatment period. A higher proportion of patients with multiple hospitalizations was HIV-negative (84/145 [58%]) than HIV-positive (45/97 [40%]).

### Hospital-based incomplete treatment

The median duration of monitoring treatment doses during all hospitalizations was 331 days (IQR 187, 476) and did not differ by HIV status. Overall hospital-based percent missed doses of anti-TB drugs was highest for amikacin (18%), capreomycin (18%), PAS (17%), and kanamycin (16%) (Table 2).

**Table 2:** Percent received doses of anti-TB drugs. Summary of percent hospital-based anti-TB drug doses received categorized by drug-resistant TB regimen, in order of lowest to highest overall percent doses received, for all regimens.

Drugs included in treatment regimen ^a^	MDR-TB Regimen			XDR-TB Regimen			M/XDR-TB Regimen			All Regimens		
	Doses received (n)	Doses prescribed (n)	Doses received (%)	Doses received (n)	Doses prescribed (n)	Doses received (%)	Doses received (n)	Doses prescribed (n)	Doses received (%)	Total doses received (n)	Total doses prescribed (n)	Total doses received (%)
Amikacin	125	131	**95.4**	30	42	**71.4** ^b^	451	566	**79.7**	606	739	**82**
Capreomycin	-	-	**-**	14,530	17,786	**81.7**	18,790	22,735	**82.7**	33,320	40,521	**82.2**
PAS	-	-	**-**	38,132	46,515	**82**	56,176	67,103	**83.7**	94,308	113,618	**83**
Kanamycin	2,269	2,710	**83.7**	-	-	**-**	8,942	10,660	**83.9**	11,211	13,370	**83.9**
Rifampicin	-	-	**-**	-	-	**-**	87	99	**87.9**	87	99	**87.9**
Augmentin	-	-	**-**	7,500	8,558	**87.6**	15,473	17,310	**89.4**	22,973	25,868	**88.8**
Clarithromycin	-	-	**-**	6,023	6,687	**90.1**	10,625	11,928	**89.1**	16,648	18,615	**89.4**
Streptomycin ^a^	-	-	-	-	-	-	194	216	89.8	194	216	**89.8**
Amoxicillin	-	-	**-**	4,589	5,030	**91.2**	9,729	10,892	**89.3**	14,318	15,922	**89.9**
Azithromycin	-	-	**-**	-	-	**-**	489	538	**90.9**	489	538	**90.9**
Dapsone	-	-	**-**	1,374	1,497	**91.8**	4,868	5,309	**91.7**	6,242	6,806	**91.7**
Ethionamide	3,268	3,399	**96.2**	17,805	19,624	**90.7**	37,323	40,248	**92.7**	58,396	63,271	**92.3**
Ofloxacin	2,614	2,751	**95**	8,586	9,414	**91.2**	22,990	24,814	**92.7**	34,190	36,979	**92.5**
Clofazimine	6	7	**85.7** ^a^	10,962	11,783	**93**	11,738	12,718	**92.4**	22,706	24,508	**92.6**
Isoniazid ^c^	221	227	**97.4** ^a^	12,443	13,423	**92.7**	17,945	19,383	**92.6**	30,609	33,033	**92.7**
Terizidone	8,103	8,605	**94.2**	40,400	43,839	**92.2**	16,750	17,657	**94.9**	65,253	70,101	**93.1**
Ethambutol	2,169	2,225	**97.5**	19,018	20,608	**92.3**	34,335	36,802	**93.3**	55,522	59,635	**93.1**
Moxifloxacin	605	636	**95.1**	13,299	14,269	**93.2**	18,783	20,150	**93.2**	32,687	35,055	**93.2**
Rifafour ^a^	21	21	**100**	87	91	**95.6**	897	964	**93.1**	1,005	1,076	**93.4**
Pyrazinamide	3,138	3,227	**97.2**	22,665	24,494	**92.5**	44,105	46,985	**93.9**	69,908	74,706	**93.6**
Levofloxacin	-	-	**-**	890	969	**91.9**	425	433	**98.2**	1,315	1,402	**93.8**
Delamanid	-	-	**-**	-	-	**-**	185	192	**96.4**	185	192	**96.4**
Linezolid	-	-	**-**	55	56	**98.2**	462	475	**97.3**	517	531	**97.4**
Bedaquiline	-	-	**-**	248	237	**104.6**	102	102	**100**	350	339	**103.2**

^a^ Some treatment regimens included anti-TB drugs recommended for DS-TB or non-WHO recommended DR-TB drugs.

^b^ In the Western Cape Province, South Africa, capreomycin is the preferred second-line injectable prescribed as part of an XDR-TB regimen.

^c^ Dose corresponding to high-dose isoniazid prescription.

**Fig 2:**
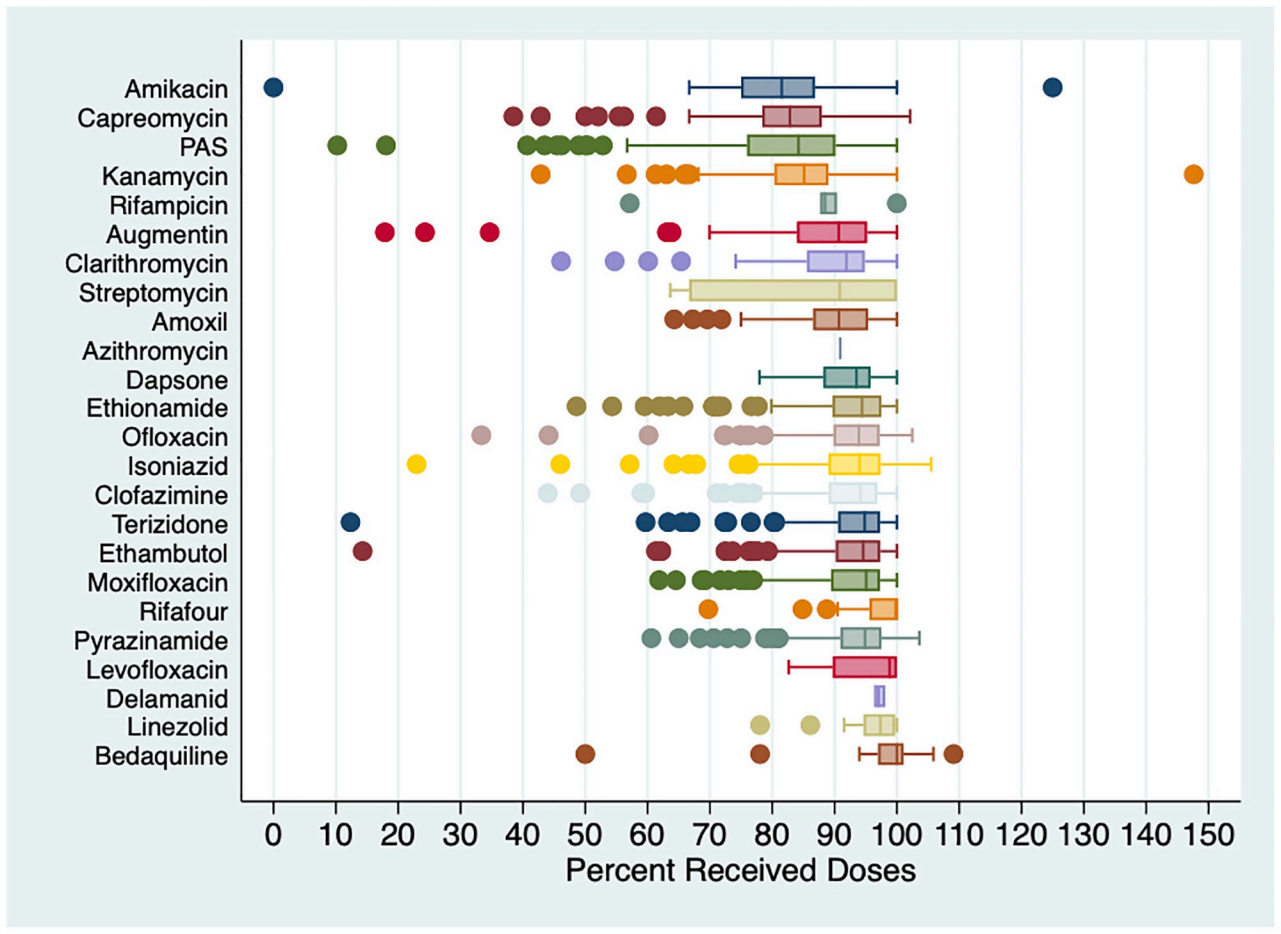
Percent received doses of anti-tuberculosis drugs. Box plots of percent of doses received per patient to anti-tuberculosis drugs during all hospitalizations according to prescription chart records, ordered from lowest to highest median percentage. Values exceeded 100% when number of doses received was greater than number of doses prescribed.

Fig 2 shows the median and distribution of doses received per patient by anti-TB drug. For 27 out of 4,336 individual drug prescription courses, the number of doses received exceeded the number of doses prescribed. When all doses received and prescribed were totaled to determine overall number of doses received per anti-TB drug, the overall percent doses received for bedaquiline was >100%.

A generalized logistic mixed effects model showed that odds of receiving doses (vs. missing doses) of clofazimine, ethambutol, ethionamide, isoniazid, moxifloxacin, ofloxacin, pyrazinamide, and terizidone were significantly higher than odds of receiving doses of capreomycin (all with P<0.001), but not kanamycin or PAS. The odds of receiving doses (vs. missing doses) of amikacin were significantly lower than odds of receiving doses of capreomycin (P<0.001). The odds of receiving doses did not vary by HIV status (OR 0.91; 95% CI 0.76, 1.08). Number of hospitalizations was not significantly associated with received doses of any anti-TB drug (P>0.09 for all drugs; S1 Fig in S1 File).

In a sensitivity analysis in which we added the remainder of the intended treatment duration to the doses prescribed for drugs that were discontinued due to adverse events we found that percent doses received substantially decreased for several drugs (S2 Table in S1 File). Among drugs prescribed for more than 10,000 doses, we found that percent doses received decreased the most for kanamycin (decreased from 84% to 76%), capreomycin (decreased from 82% to 76%), ethionamide (decreased from 92% to 87%), and isoniazid (decreased from 93% to 90%). Among drugs prescribed for fewer than 10,000 doses, amikacin (decreased from 82% to 62%) and dapsone (decreased from 92% to 83%) had the most notable changes.

### Reason for discontinuation of treatment

Reasons for discontinuation of a medication were considered separately from reasons for missed doses. Treatment failure, death, treatment interruption during hospitalization, adverse events, and patient refusal or absconding from hospital were considered unfavourable reasons for discontinuation. Together, these were responsible for nearly half (47%) of all reasons for treatment discontinuation, although reasons varied widely per drug (Table 3, S3 Table in S1 File).

**Table 3:** Reasons for discontinuation of anti-TB drugs. Unfavorable reasons drug-resistant tuberculosis patients discontinued anti-tuberculosis drugs while hospitalized as reported in medical records.

Drugs included in regimen	Number of patients who received drug	Cumulative treatment duration (years)	Treatment discontinuation for any unfavorable reason (Number of events per year) ^a^	Unfavorable Reasons for discontinuation of anti-TB drug (Number of events per year) ^a^ ^b^					Other reasons ^a^ ^e^
				Abscond or refuse treatment	Adverse event ^c^	Death	Treatment failure ^c^	Treatment interruption ^d^	
Amikacin	20	2.9	2.38	0.68	1.02	0.68	0	0	4.76
Amoxil	50	24.2	1.20	0.21	0	0.29	0.29	0.41	1.49
Augmentin	77	40.2	1.64	0.32	0.05	0.27	0.45	0.55	1.32
Azithromycin	1	1.6	0.00	0	0	0	0	0	0.62
Bedaquiline	21	6.8	1.17	0.29	0.15	0.29	0	0.44	2.48
Capreomycin	211	145.4	2.04	0.4	0.56	0.13	0.41	0.54	0.72
Clarithromycin	55	27.9	1.87	0.47	0.04	0.25	0.5	0.61	1.11
Clofazimine	114	74.8	1.90	0.44	0.08	0.37	0.4	0.6	0.67
Dapsone	40	19	1.79	0.32	0.11	0.32	0.69	0.37	1.16
Delamanid	3	1.2	0.81	0	0	0	0	0.81	2.44
Ethambutol	221	183.7	1.46	0.44	0.14	0.16	0.29	0.43	0.97
Ethionamide	234	191.6	1.61	0.51	0.26	0.18	0.22	0.45	0.98
Isoniazid	156	102	1.69	0.39	0.17	0.24	0.37	0.52	0.86
Kanamycin	137	47.1	2.10	0.47	0.7	0.13	0.42	0.38	2.04
Levofloxacin	15	4.9	0.81	0.4	0	0	0	0.4	2.63
Linezolid	18	6.1	1.65	0.17	0.49	0.33	0	0.66	2.3
Moxifloxacin	137	107.2	1.67	0.45	0.05	0.32	0.35	0.51	0.7
Ofloxacin	153	115.3	1.06	0.32	0.03	0.12	0.23	0.35	1.31
PAS	217	172.5	1.64	0.57	0.08	0.18	0.35	0.46	0.67
Pyrazinamide	238	230.8	1.44	0.43	0.09	0.21	0.26	0.45	0.82
Rifafour	6	1.9	0.54	0	0.54	0	0	0	4.82
Rifampicin	2	0.38	0.00	0	0	0	0	0	8
Streptomycin	2	0.61	1.65	1.65	0	0	0	0	4.94
Terizidone	235	213.3	1.54	0.43	0.08	0.22	0.32	0.49	0.74

^a^ Columns are color scaled from lowest (white) to highest (red).

^b^ Reasons for discontinuation of drugs were reported if patients received anti-TB drugs for ≥1 day. Reasons for same day prescription and discontinuation of anti-TB drugs were not reported (e.g., adverse event, death, refusal of treatment).

^c^ As stated on prescription chart or in patient medical record by physician who discontinued the drug.

^d^ Stop taking medication >7 days, initiated by patient or provider.

^e^ Other reasons shown in S3 Table in S1 File and include: Course of treatment completed, changed to another anti-TB drug, continued as community-based treatment, transferred to another facility or province, granted leave from hospital (missed doses unknown), treatment continued beyond censor date, uncategorized, and unknown.

Drug discontinuation due to adverse events was highest for amikacin (1.02 events per year follow-up), kanamycin (0.7 events per year follow-up), and capreomycin (0.56 events per year follow-up). Rifafour and linezolid also had relatively high rates of discontinuation due to adverse events, though these were infrequently prescribed during our study. The highest rates of discontinuation due to patient refusal or absconding, among frequently prescribed drugs, were for amikacin (0.68 events per year follow-up) and PAS (0.57 events per year follow-up). Streptomycin had the highest discontinuation rate (1.65 events per year follow-up) but was only prescribed to 2 study patients.

### Factors associated with completion of PAS

Among the anti-TB drugs with the highest percent missed doses, PAS is the only oral drug included in the most recent WHO MDR-TB treatment recommendations list (Group C) [32]. A cumulative probability model showed that after adjusting for sex, age at first DR-TB admission, race, and HIV status, the odds of completion of PAS were 1.87 times higher for individuals without previous TB than those who had previous drug-susceptible TB (95% CI 1.10, 3.18; p = 0.02). Age at first DR-TB admission, sex, race, and HIV status were not associated with completion of PAS.

### Data quality control

To verify the impact of discrepant health professional documentation on recorded research data and to verify data captured by novice data capturers we did in-depth data quality control for 134/242 (55%) study patients. No errors affecting treatment data reporting or study outcomes were noted in 114/134 (85%) cases. In the 20 cases where treatment data, as recorded by researchers, were revised, two were due to updated study specific standard operating procedures, three due to data capture errors and 15 due to incorrect treatment dose counting by data capturers.

## Discussion

In this study we found that hospital-based incomplete treatment with individual anti-TB drugs varied considerably. Overall missed doses of PAS and the second-line injectable drugs (capreomycin, kanamycin, and amikacin) were higher (16–18%) than the other anti-TB drugs commonly included in DR-TB regimens. Doses received of other most commonly used anti-TB drugs exceeded 92%. PAS and second-line injectable drugs were considered core drugs in MDR and XDR regimens during the study period.

Efforts to eliminate injectable drugs from DR-TB treatment regimens are supported in the 2020 updated WHO guidelines on DR-TB, with recommendations for all-oral shorter and longer regimens [32]. However, for patients treated with WHO-recommended longer regimens whose treatment regimens cannot be entirely composed of Group A and B drugs, amikacin remains an option for treatment in Group C. Similarly, PAS is also retained as a Group C drug. In some regions of the world, health system failures such as limited access to bedaquiline or high background rates of resistance to key DR-TB treatment drugs such as fluoroquinolones [33] may still necessitate the incorporation of amikacin or PAS in treatment regimens. Our findings of high percent missed doses and frequencies of discontinuation due to adverse events and patient refusal suggest that in such cases, treatment monitoring and support with particular attention to these drugs may be warranted, even in hospital settings.

Because PAS is an oral medication that may still be used in some WHO-recommended longer regimens, we assessed factors associated with complete treatment of PAS and found that previous drug-susceptible TB was independently associated with lower completion of PAS. Similarly, Batte and colleagues found that previous TB was associated with overall poor adherence to MDR-TB treatment at a referral hospital in Uganda [34]. The reasons for the association of decreased adherence to treatment with prior TB are unclear. PAS has a high frequency of reported gastrointestinal intolerance and lower reported rates of reversible hypothyroidism and liver toxicity [35]. It is possible that PAS intolerance or toxicity are enhanced when used in combination or after treatment with other drugs, [36] though we are unable to verify this with our data. In this study reasons for discontinuing PAS were predominantly recorded as patient refusal rather than adverse events. Accordingly, missed doses of PAS did not decrease substantially in a sensitivity analysis of missed doses of drugs discontinued due to adverse events. Additional studies regarding reasons for patient refusal of PAS and how these relate to criteria for recording adverse events may provide further insight into incomplete treatment of PAS. We also considered whether treatment fatigue could play a role in incomplete treatment but did not find significantly increased percent missed doses of individual drugs according to number of hospitalizations in our study cohort.

The high percent missed doses of amikacin and PAS may have been related in part to continuation of prescribed doses of these drugs despite repeated patient refusal. If prescriptions for these drugs would have been discontinued earlier, upon patient refusal, duration of recorded amikacin and PAS treatment would have been shorter and the percent missed doses would have been lower. Adjustments to treatment regimens due to patient refusal of drugs may have facilitated a more patient-centered approach to treatment. However, limited availability of viable treatment alternatives during the study period may have contributed to extended treatment periods with PAS despite frequent patient refusal.

The higher percent missed doses of PAS and injectable drugs did not differ according to HIV status. We were unable to compare incomplete treatment of ART and anti-TB drugs due to differences in ART medication capture in hospital records. However, adherence to XDR-TB treatment was found to be significantly lower than adherence to ART in a previous study in South Africa [37].

To our knowledge, this is the first study to determine hospital-based incomplete treatment of individual DR-TB drugs in the Africa region. Wang and colleagues assessed treatment interruption in MDR-TB patients in China using hospital records and self-report [10]. Consistent with our study findings of high percent missed doses of amikacin, they found that amikacin was the drug with the most frequent interruptions, including both short (1–14 days non-receipt of drug) and long (>14 days non-receipt of drug) interruptions. Although we found that percent missed doses of amikacin was higher when prescribed during treatment courses for XDR-TB or M/XDR-TB, compared to MDR-TB only, relatively few patients were treated with amikacin, and this usually occurred when kanamycin was contraindicated. These factors limit conclusions that can be drawn about optimal populations for use of amikacin.

Prior to decentralization of DR-TB care in South Africa, hospital-based DR-TB treatment was routinely done for many reasons, including to control and oversee administration of expensive medications, allow for monitoring of adverse drug events, and increase treatment adherence in general [38]. Several studies have shown many advantages of community-based care over centralized care models for DR-TB, including improved treatment success [38,39]. Even after decentralization of DR-TB care in South Africa in 2011, some previously non-adherent DR-TB patients have been hospitalized to support adherence [24].

Anti-TB drug adherence, particularly hospital-based adherence, relies on multiple interactive factors including patient perceptions and behavior, the extent of multi-disciplinary teamwork, medication supply chain, education and training of staff, and regular quality improvement of adherence support [40,41]. Furthermore, acquired anti-TB drug resistance and previous poor adherence could lead to use of less tolerable anti-TB drugs, suggesting that both patient factors and characteristics of the individual drugs may contribute to observed adherence. Underlying challenges in high-volume, resource-limited settings may further impact patient services and negatively impact completion of treatment, including patient adherence and other factors such as interrupted drug supply, that contribute to missed doses [42].

During our medication chart review we at times noted medication record discrepancies such as implausible medication administration (e.g., doses given when patients were on approved weekend leave from hospital) and differences between physician prescription orders and prescription information recorded on prescription charts. Support for accurate prescription chart documentation, as a health systems improvement measure, may assist optimizing assessment of patient completion of treatment. Evidence of incomplete treatment is critical for optimal patient care, since it allows clinical decisions such as change of regimen and support for continuation of care.

The definition of adherence is generally limited to completeness of treatment related to active patient choices, though may more broadly include other reasons for missed doses, such as specified lab abnormalities in clinical trials [17]. Because we were unable to distinguish patient choices from other reasons for missed doses consistently, we generally avoided the use of the term adherence. We were also unable to distinguish between missed doses due to refusal, intolerance, or adverse effects. Our separate assessment of reasons for treatment discontinuation during hospitalization, however, suggests that the second-line injectable drugs and linezolid were discontinued due to adverse effects more frequently than other anti-TB drugs, consistent with known toxicity of these drugs [43]. We used available data to calculate percent missed doses and were unable to characterize more complex patterns of incomplete treatment (or non-adherence), including the timing and intermittency of missed doses [44]. Future studies may help determine the importance of timing and frequency of missed doses on DR-TB treatment choices and outcomes.

The high overall mortality in our study cohort [45] is similar to studies conducted before the widespread use of bedaquiline in treatment regimens in South Africa among patients with highly drug-resistant disease [5,46]. Although we did not find that percent missed doses increased per hospitalization for individual drugs, those with frequent missed doses may not have survived to have multiple hospitalizations.

When medications that are frequently refused or poorly tolerated by patients need to be included in treatment regimens (e.g., amikacin and PAS, which are still options in the 2020 WHO treatment guidelines), approaches to improve patient completion of treatment and ensure reliable recording of treatment data that are more effective and less restrictive to patients should be considered. For example, implementation of digital technologies that can monitor and remind patients to take medication have the potential to be more patient-centered and successful [47,48]. The implementation of such technologies in TB high burden settings will need to take patient perceptions of TB, adherence support technologies and adherence definitions used into account [44,49]. Furthermore, thoughtful timing of incorporation of such drugs in treatment regimens to maximize their usefulness in the regimen before they potentially need to be discontinued should be considered. Ultimately, attention should be given to inclusion of well-tolerated anti-TB medications when developing treatment regimens whenever possible. Although delamanid, linezolid, and bedaquiline appeared promising in terms of in-hospital completion of treatment, few patients included in our cohort were treated with these medicines since these drugs were just being implemented during our study period.

### Limitations

Our study had several limitations. The patients in our cohort were not representative of all DR-TB patients. Patients were selected based on serial drug-susceptibility testing, were more likely to be sicker and to have had previous exposure to first and second-line drugs. Furthermore, after decentralization and deinstitutionalization of DR-TB treatment, patients were mainly admitted to the hospital due to severity of illness. All these factors may have been associated with prior non-completion of treatment and missed doses during hospitalization. Our study patients may have been less likely to complete treatment with anti-TB drugs, even in the hospital setting, than other DR-TB patients. On the other hand, the severity of illness may have prompted patients to be more careful in taking their medication. Further studies, including qualitative assessments of patient and healthcare worker experience of completion of treatment, and evaluation of adherence support mechanisms, may help improve understanding of inpatient incomplete treatment.

Our analyses were primarily descriptive, particularly in reporting in-hospital incomplete treatment. The retrospective nature of the dataset prevented more inferential analyses that accounted for treatment after hospital discharge, and our results should be interpreted only in terms of medication completion during hospitalization.

Our analysis of drug incomplete treatment was limited by what was recorded on prescription charts. We investigated and corrected errors found during data quality checks but were not able to resolve all apparent prescription chart errors. Since we captured reasons for discontinuation of drugs as recorded in the prescription charts and medical records, we could have misclassified reasons for discontinuation. For example, a drug may have been recorded as having been discontinued due to treatment interruption when it was truly discontinued due to an adverse event. Focused pharmacovigilance studies on hospitalized DR-TB patients may provide additional insight into the relationship between treatment completion, drug tolerability, and adverse events. Healthcare provider education and training on the impact of accurate documentation on patient care and on research is vital [50].

We did not have access to anti-TB drug treatment data from all hospitals where patients received DR-TB treatment, including missing prescription charts during hospitalizations. Excluding periods when patients did not receive a drug for >7 consecutive days from missed dose calculations could have resulted in underestimation of percent missed doses. Prospective hospital and community-based anti-TB drug adherence studies, including the use of improved adherence monitoring technology, is essential given the change to newer anti-TB drugs and shorter duration of DR-TB treatment.

### Conclusion

Incomplete DR-TB treatment was not uniform during hospitalization, with percent missed doses to amikacin, kanamycin, capreomycin, and PAS higher than other anti-TB drugs. Our study was unable to distinguish whether missed doses occurred due to poor tolerability of anti-TB drugs or patient-driven factors. However, we found that the injectable drugs were discontinued frequently due to adverse effects and both amikacin and PAS were frequently discontinued due to patient refusal. Patient-centered approaches to adherence monitoring and treatment support are particularly warranted when drugs with better completion and tolerability than amikacin and PAS (2020 WHO Group C drugs) are unable to be used.

## Supporting information

S1 File This file contains S1–S3 Tables, S1 Fig and analysis code. (PDF)

S2 File Supplement for publication—general study data. (CSV)

S3 File Supplement for publication—medication count data. (CSV)

S4 File Supplement for publication—completion per Hospitalization. (CSV)

S5 File Supplement for publication—hospitalization data. (CSV)

S6 File Supplement for publication—PAS model. (CSV)

S7 File Supplement for publication—reason for discontinuation. (CSV)
