# Supplementary material for: Variation in missed doses and reasons for discontinuation of anti-tuberculosis drugs during hospital treatment for drug-resistant tuberculosis in South Africa
Source: PLoS One. 2023 Feb 13;18(2):e0281097. doi: 10.1371/journal.pone.0281097 (PMC9925007; doi:10.1371/journal.pone.0281097)
Supplement: S1 File — (PDF) [file pone.0281097.s001.pdf]

SUPPORTING INFORMATION

Variation in missed doses and reasons for discontinuation of anti-tuberculosis drugs during hospital treatment for drug-resistant tuberculosis in South Africa

Elize Pietersen, Kim Anderson, Helen Cox, Keertan Dheda, Aihua Bian, Bryan E. Shepherd, Timothy R. Sterling, Robin M. Warren, Yuri F. van der Heijden

Contents

S1 Table

S2 Table

S3 Table

S1 Figure

Analysis Code

Data Files (See separate .csv files):

- Supplement for Publication – General study data.csv
- Supplement for Publication – Medication count data.csv
- Supplement for Publication – Completion per Hospitalization.csv
- Supplement for Publication – Hospitalization data.csv
- Supplement for Publication – PAS model.csv
- Supplement for Publication – Reason for discontinuation.csv

S1 Table. Standardized Treatment Regimens. Recommended standardized regimens during study period, including duration, for treatment of MDR-TB and XDR-TB according to South African Department of Health policy guidelines, 2011. Patients with previous exposure to second-line anti-tuberculosis drugs required individualized treatment regimens. Drugs that differ between MDR-TB and XDR-TB treatment regimens are bolded.

Treatment phase	Duration	Drugs included in treatment regimen	
		MDR-TB regimen	XDR-TB regimen
Intensive phase	6 months	**Kanamycin or Amikacin**<br>Moxifloxacin<br>Ethionamide<br>Terizidone<br>Pyrazinamide	**Capreomycin**<br>Moxifloxacin<br>Ethionamide<br>Terizidone<br>Pyrazinamide<br>**PAS**<br>**Clofazimine**
Continuation phase	≥18 months after TB culture conversion	Moxifloxacin<br>Ethionamide<br>Terizidone<br>Pyrazinamide	Moxifloxacin<br>Ethionamide<br>Terizidone<br>Pyrazinamide<br>**PAS**<br>**Clofazimine**
Additional second-line drugs to consider		Ethambutol<br>Levofloxacin<br>Prothionamide<br>Cycloserine<br>Clofazimine<br>Amoxicillin-clavulanate<br>Clarithromycin<br>Azithromycin<br>Linezolid<br>Thioacetazone<br>Imipenem<br>Isoniazid (high dose)	
Other drugs post 2011 Guidelines		Bedaquiline<br>Delamanid<br>Pretomanid	

S2 Table. Comparison of hospital-based doses received of anti-tuberculosis drugs based on primary analysis and a sensitivity analysis in which we added the remainder of the intended treatment duration to the doses prescribed for drugs that were discontinued due to adverse events.

Drugs included in treatment regimen [a]	All Regimens			Sensitivity Analysis		
	Total doses received (n)	Total doses prescribed (n)	Total Doses received (%)	Total doses received (n)	Total doses prescribed (n)	Total Doses received (%)
Amikacin	606	739	**82**	606	986	**61.5**
Capreomycin	33,320	40,521	**82.2**	33,320	44,120	**75.5**
PAS	94,308	113,618	**83**	94,308	114,294	**82.5**
Kanamycin	11,211	13,370	**83.9**	11,211	14,719	**76.2**
Rifampicin	87	99	**87.9**	87	99	**87.9**
Augmentin	22,973	25,868	**88.8**	22,973	26,042	**88.2**
Clarithromycin	16,648	18,615	**89.4**	16,648	18,749	**88.8**
Streptomycin [a]	194	216	**89.8**	194	216	**89.8**
Amoxicillin	14,318	15,922	**89.9**	14,318	15,922	**89.9**
Azithromycin	489	538	**90.9**	489	538	**90.9**
Dapsone	6,242	6,806	**91.7**	6,242	7,540	**82.8**
Ethionamide	58,396	63,271	**92.3**	58,396	66,990	**87.2**
Ofloxacin	34,190	36,979	**92.5**	34,190	37,036	**92.3**
Clofazimine	22,706	24,508	**92.6**	22,706	24,702	**91.9**
Isoniazid [b]	30,609	33,033	**92.7**	30,609	34,073	**89.8**
Ethambutol	55,522	59,635	**93.1**	55,522	61,534	**93**
Terizidone	65,253	70,101	**93.1**	65,253	70,497	**92.6**
Moxifloxacin	32,687	35,055	**93.2**	32,687	35,132	**93**
Rifafour [a]	1,005	1,076	**93.4**	1,005	1,106	**90.1**
Pyrazinamide	69,908	74,706	**93.6**	69,908	75,604	**92.5**
Levofloxacin	1,315	1,402	**93.8**	1,315	1,402	**93.8**

Delamanid	185	192	**96.4**	185	192	**96.4**
Linezolid	517	531	**97.4**	517	531	**97.4**
Bedaquiline	350	339	**103.2**	350	339	**103.2**

[a] Some treatment regimens included anti-TB drugs recommended for DS-TB or non-WHO recommended DR-TB drugs

[b] Dose corresponding to high-dose isoniazid prescription

S3 Table. Other Reasons for discontinuation of Drugs. Expanded categories of "Other reasons" (**Table 3**, main paper) drug-resistant tuberculosis patients discontinuation of anti-tuberculosis drugs while hospitalized

Drugs included in treatment regimen	Number of patients who received drug	Cumulative treatment duration (years)	Reasons for discontinuation of anti-TB drug (Number of events per year) [a, b]							
			Change of medication prescribed	Treatment continued after discharge	Course of treatment complete	Granted leave [c]	Transfer out	Treatment beyond censor date	Not Categorized [d]	Unknown
Amikacin	20	2.9	2.72	0	1.02	0	0.68	0	0	0.34
Amoxicillin	50	24.2	0	0.33	0.54	0.04	0.04	0	0.04	0.5
Augmentin	77	40.2	0	0.27	0.35	0.02	0.05	0	0.12	0.5
Azithromycin	1	1.6	0	0.62	0	0	0	0	0	0
Bedaquiline	21	6.8	0	1.17	0.88	0	0	0.15	0.15	0.15
Capreomycin	211	145.4	0.08	0.1	0.25	0.06	0.03	0	0.06	0.14
Clarithromycin	55	27.9	0	0.18	0.14	0	0.11	0	0.04	0.65
Clofazimine	114	74.8	0.04	0.43	0	0	0.01	0.03	0.07	0.09
Dapsone	40	19	0	0.47	0.05	0.05	0.05	0	0.11	0.42
Delamanid	3	1.2	0	0.81	1.63	0	0	0	0	0
Ethambutol	221	183.7	0.04	0.58	0.05	0.05	0.07	0	0.03	0.16
Ethionamide	234	191.6	0.05	0.67	0.03	0.03	0.07	0	0.04	0.09
Isoniazid	156	102	0.04	0.51	0.05	0.04	0.06	0.01	0.01	0.15
Kanamycin	137	47.1	0.62	0.93	0.3	0	0.13	0	0.02	0.04
Levofloxacin	15	4.9	1.01	1.01	0	0	0	0.4	0	0.2
Linezolid	18	6.1	0	1.81	0.33	0	0	0.16	0	0
Moxifloxacin	137	107.2	0.08	0.51	0.01	0.01	0.04	0	0.01	0.04
Ofloxacin	153	115.3	0.32	0.75	0	0.07	0.08	0	0.01	0.08

PAS	217	172.5	0.02	0.45	0.02	0.04	0.04	0.01	0.02	0.06
Pyrazinamide	238	230.8	0.02	0.62	0.02	0.03	0.06	0.01	0.02	0.05
Rifafour	6	1.9	1.61	1.07	1.07	0	0.54	0	0	0.54
Rifampicin	2	0.38	0	2.67	2.67	0	2.67	0	0	0
Streptomycin	2	0.61	0	3.29	0	0	0	0	1.65	0
Terizidone	235	213.3	0.02	0.58	0.005	0.04	0.04	0.005	0.02	0.02

[a] Columns are color scaled from lowest (white) to highest (red)

[b] Reasons for discontinuation of drugs were reported if patients received anti-TB drugs for ≥1 day. Reasons for same day prescription and discontinuation of anti-TB drugs were not reported (e.g., adverse event, death, refusal of treatment).

[c] Usually 2 weekend days or 2 weeks during festive seasons, as authorized by a physician

[d] Not categorize d included reasons not otherwise reported (e.g., pregnancy, erroneous administration, conflicting chart details).

S1 Figure. **Percent received doses of select anti-TB drugs per hospitalization.** Boxplot of percent doses received per hospitalization of selected core anti-TB regimen drugs with highest overall percent missed doses: capreomycin, kanamycin, and PAS. Moxifloxacin was the only drug with P<0.25. Amikacin had substantially fewer prescribed doses during study period.

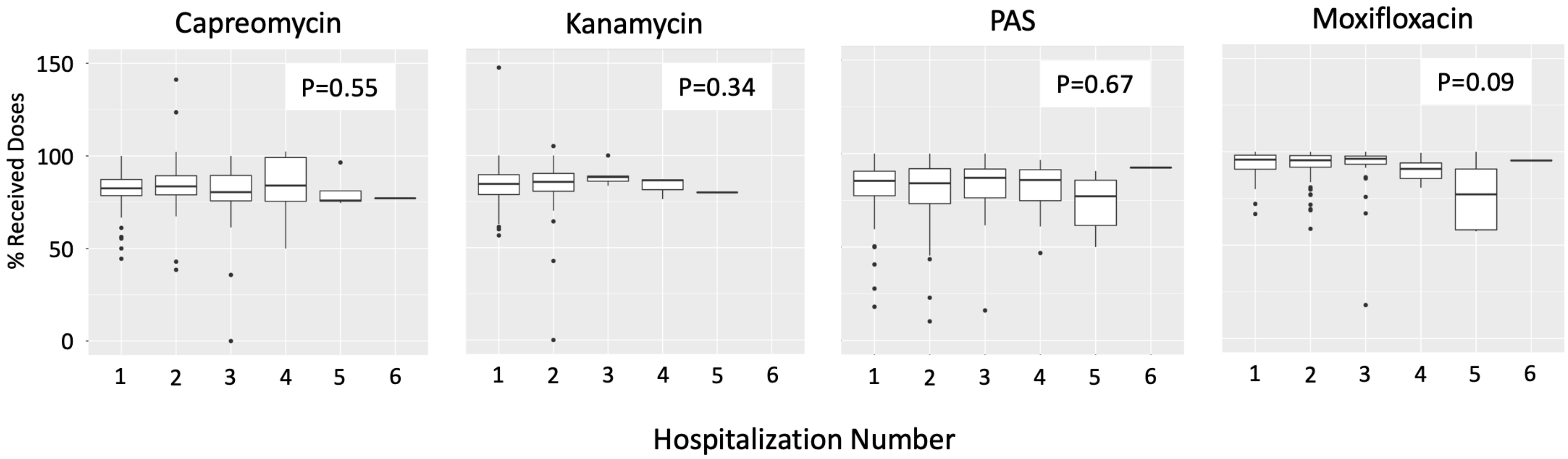

Analysis Code:

Table 1 (STATA):

```
sum Ageatfirstadmission, detail

bysort HIVStatus: sum Ageatfirstadmission, detail

ranksum Ageatfirstadmission, by(HIVStatus)

tab Gender

tab Gender HIVStatus, col row mi chi2

tab race_mod

tab race_mod, nolabel

tab race_mod HIVStatus, col row mi chi2

tab Smokingever

tab Smokingever HIVStatus, col row mi chi2

tab smoke_ever_mod if smoke_ever_mod != 9

tab smoke_ever_mod HIVStatus if smoke_ever_mod != 9, col row mi chi2

tab AnySubstancesever

tab AnySubstancesever HIVStatus, col row mi chi2

tab subs_ever_mod if subs_ever_mod != 9

tab subs_ever_mod HIVStatus if subs_ever_mod != 9, col row mi chi2

sum Weightatfirstadmission, detail

bysort HIVStatus: sum Weightatfirstadmission, detail

ranksum Weightatfirstadmission, by(HIVStatus)

tab edu

tab edu HIVStatus, col row mi chi2

tab hiv_firstadm

tab OnARVsatfirstadmission

sum CD4at1stadm, detail

sum VLat1stadm, detail
```

```
sum VLat1stadm if VLat1stadm != 0, detail

tab CXRat1stadm

tab CXRat1stadm HIVStatus, col row chi2

sum CXRat1stadm if CXRat1stadm != ., detail

bysort HIVStatus: sum CXRat1stadm if CXRat1stadm != ., detail

ranksum CXRat1stadm, by(HIVStatus)

sum disease_scoreat1stadm, detail

bysort HIVStatus: sum disease_scoreat1stadm, detail

ranksum disease_scoreat1stadm, by(HIVStatus)

sum cavity_scoreat1stadm, detail

bysort HIVStatus: sum cavity_scoreat1stadm, detail

ranksum cavity_scoreat1stadm, by(HIVStatus)

tab PriorDSTB

tab PriorDSTB HIVStatus, col row chi2

sum hbat1stadm, detail

bysort HIVStatus: sum hbat1stadm, detail

ranksum hbat1stadm, by(HIVStatus)

sum creatineat1stadm, detail

bysort HIVStatus: sum creatineat1stadm, detail

ranksum creatineat1stadm, by(HIVStatus)

tab ChronicKidneydisease

tab ChronicKidneydisease HIVStatus, col row mi chi2

tab Diabetic

tab Diabetic HIVStatus, col row mi chi2

tab Psychiatricdisorder

tab Psychiatricdisorder HIVStatus, col row mi chi2

tab AdmissionsSingleMultiple

tab AdmissionsSingleMultiple HIVStatus, col row mi chi2
```

```
tab TBdxat1stadm
tab TBdxat1stadm HIVStatus, col row mi chi2
tab regimen_total
tab regimen_total HIVStatus, col row mi chi2
tab vital_stat_end
tab vital_stat_end HIVStatus, col row mi chi2
```

Incomplete Treatment:

Percent missed doses = (number of doses missed / number of doses prescribed) x 100

Percent received doses = (number of doses received / number of doses prescribed) x 100

Rate of discontinuation of individual drugs (STATA):

```
gen rate_yrs = n_reason_drug/duration_drug_years
```

Generalized logistic mixed effects model to estimate adjusted odds ratios and 95% confidence intervals of receiving a drug (versus missing a drug) during hospitalization (STATA):

```
meqrlogit recmis i.drug_ultrashort hivstatus || StudyNumber: , or
```

Factors Associated with completion of PAS treatment (R statistical software):

```
mod3<-orm(adher_PAS ~ sex + prevtb + Ageatfirstadmission + hiv_firstadm +
race_mod, data=d, x=TRUE, y=TRUE)
summary(mod3, Ageatfirstadmission=c(20,30))
anova(mod3)
```
